# Biocompatibility evaluation of peo-treated magnesium alloy implants placed in rabbit femur condyle notches and paravertebral muscles

**DOI:** 10.1186/s40824-022-00279-1

**Published:** 2022-07-06

**Authors:** Seong Ryoung Kim, Keon Mo Lee, Jin Hong Kim, Young Jin Choi, Han Ick Park, Hwa Chul Jung, Hyung Jin Roh, Jee Hye Lo Han, Joon Rae Kim, Bu-Kyu Lee

**Affiliations:** 1 Department of Oral and Maxillofacial Surgery, Yonsei University Dental Hospital, Seoul, Republic of Korea; 2 Department of Oral and Maxillofacial Surgery, College of Medicine, University of Ulsan, Asan Medical Center, Seoul, Republic of Korea; 3 R&D Division, U&I Corporation, Uijongbu, 480-050 Republic of Korea; 4 2nd Analysis Lab, 127, Mapo-daero, Mapo-gu, Seoul, Republic of Korea

**Keywords:** New Zealand rabbit, Biocompatibility, Magnesium, PEO (plasma electrolytic oxidation), Hydrogen gas, Mg-1wt%Zn-0.1wt%ca alloy

## Abstract

**Background:**

Magnesium alloys have been receiving much attention for use in biodegradable metal implants because of their excellent mechanical properties and biocompatibility. However, their rapid breakdown and low bioactivity can cause the implant to lose mechanical integrity before the bone is completely healed. Moreover, hydrogen gas released during degradation can significantly delay the tissue regeneration process. To solve the instability of magnesium alloys, Zn and Ca can be added to improve the mechanical properties and biocompatibility. One other way to improve the mechanical properties of Mg is plasma electrolytic oxidation (PEO), which provides a dense, thick ceramic-like coating on the Mg surface. In this study, high-purity Mg was selected as the control, and Mg-1wt%Zn-0.1wt%Ca alloy and PEO-treated Mg-1wt%Zn-0.1wt%Ca alloy were selected as the test materials; the results of radiographic and histological analyses of their biocompatibility are reported herein.

**Materials and method:**

Nineteen New Zealand white rabbits were used in the study. Rod-bars (Ø2.7 × 13.6 mm) were placed on both paravertebral muscles, and cannulated screws (Ø2.7x10mm) were placed on both femur condyle notches. Each animal was implanted in all four sites. X-rays were taken at 0, 2, 4, 8, and 12 weeks, micro-CT, and live-CT were taken at 4, 8, and 12 weeks. At weeks 4, 8, and 12, individuals representing each group were selected and sacrificed to prepare specimens for histopathological examination.

**Result:**

The results confirm that in vivo, Mg-1wt%Zn-0.1wt%Ca alloy had higher corrosion resistance than high-purity Mg and safely degraded over time without causing possible side effects (foreign body or inflammatory reactions, etc.). In addition, PEO treatment of Mg-1wt%Zn-0.1wt%Ca alloy had a positive effect on fracture recovery by increasing the bonding area with bone.

**Conclusion:**

Our results suggest that PEO treatment of Mg-1wt%Zn-0.1wt%Ca alloy can be a promising biomaterials in the field of various clinical situations such as orthopedic and maxillofacial surgerys.

**Supplementary Information:**

The online version contains supplementary material available at 10.1186/s40824-022-00279-1.

## Background

Ti and stainless-steel internal fixation devices have been the gold-standard for orthognathic surgery and for repairing craniofacial fractures [1]. However, these materials can lead to long-term complications such as tissue irritation, infection, radiographic image interference, skeletal growth interference (especially pediatrics), aesthetically undesirable features (mainly craniofacial implants), and thermal sensitivity, as well as the potential requirement of a second surgery for removal of the fixation material [2–6].

Recently, magnesium alloys have been receiving much attention for biodegradable metal implants because of their excellent mechanical properties and biocompatibility [7–9]. Magnesium ion, which occurs when the Mg alloy is degraded, strengthens bone healing and promotes new bone formation [10–12]. Recent studies have uncovered the mechanism by which magnesium ions activate canonical Wnt signaling by inducing osteogenic activity in the bone marrow space [13]. Although Mg has two-thirds of the strength of Al and only a quarter of that of Fe, it also has high thermal conductivity, high dimensional stability, excellent electromagnetic shielding properties, high attenuation characteristics, excellent machining properties, and is easily recycled [14]. Magnesium alloys can reduce or avoid the “stress-shielding” effect of bone tissue due to their close elastic modulus values [6, 15]. Furthermore, because of the biodegradability of Mg, re-operation for implant removal can be avoided [16]. Mg has many beneficial properties, but its rapid breakdown and low bioactivity can cause an implant to lose mechanical integrity before the bone has completely healed [16–18]. In particular, the tissue regeneration process can be significantly delayed by gas released during degradation [19]. Overall, Mg degrades in vivo via the corrosion reaction;

Mg + 2H_2_O - > Mg (OH)_2_ + H_2_,(Under standard pressure and ambient temperature conditions) which shows that 1 g of pure Mg produces about 1 L of H_2_, which can accumulate in tissue cavities [12, 20]. Although small gas cavities have little effect on biosystems because the gas is exchanged quickly in the surrounding tissue, [21] the effects of large ones can be harmful. According to Noviana et al. [22], excessive hydrogen gas evolution in Mg-implanted rats spreads from the muscles to the looser subcutaneous tissue, thereby causing massive subcutaneous emphysema. Moreover, it can also create pressure that causes mechanical impairment of bone regeneration, thereby leading to distinct callus formation [23]. Indeed, gas cavity formation is the main reason for discarding Mg after its initial use [24]. Furthermore, pure Mg is not suitable for clinical use because its porous structure increases the surface area and thus accelerates the rate of degradation. Therefore, the addition of other metals can effectively reduce the degradation rate of magnesium alloys to meet the actual requirement in the human body.

Adding alloying elements to improve mechanical strength and corrosion resistance is very effective. Mg-Ca alloys are not cytotoxic and accelerate the formation of new bone by gradually degrading within 90 days in vivo [25]. These alloying elements improve ductility as they are effective particle refiners for Mg [26], and the addition of Zn to an Mg-Ca binary alloy can optimize the mechanical and degradation properties [27]. Therefore, the application of Mg-Zn-Ca alloys to bioresorbable internal fixation has attracted attention in recent years [27]. Hence, in our study, 1 wt% Zn and 0.1 wt% Ca were added in the Mg melt to increase the melt fluidity and promote the mechanical properties and corrosion resistance of this porous material by as much as possible.

Plasma electrolytic oxidation (PEO), also known as micro-arc oxidation, is a promising and environmentally friendly surface treatment developed from conventional anodizing treatment that can provide a dense, thick ceramic-like coating on the Mg surface. PEO modifies the surface, which increases the initial corrosion resistance and mechanical strength of the alloy because the barrier layer protects the substrate from the surrounding biological fluids [28]. Moreover, PEO controls the porosity during the electrolytic passivation process (sparking phenomenon). Porous surfaces at the micro- or nano-level promote cell adhesion or proliferation, thereby leading to the rapid healing of local tissues [29–34]. Last, PEO-treated layers have low toxicity [35]. This process can be applied to medical devices with complex geometries such as anterior cruciate ligament screws, plates, and pins [35–38].

The aim of this study is to analyze the biocompatibility of a biodegradable magnesium alloy with and without PEO treatment.

## Materials and methods

### Alloys

In this experiment, we prepared high-purity Mg (99.99 wt%), Mg-1wt%Zn-0.1wt%Ca alloy and PEO-treated Mg-1wt%Zn-0.1 wt% Ca alloy rods and screws. The material was cast in a vacuum furnace to reduce impurities and underwent a two-stage extrusion manufacturing process and additional plastic working to improve strength. In addition, the PEO surface treatment was undertaken using a constant voltage method in a phosphate-based electrolyte. All related processes were performed by U&I Corporation (Uijeongbu-si, Gyeonggi-do, Korea).

### The in-vivo animal model

The present in-vivo animal study was conducted in accordance with international standards on animal welfare [39] and was approved by the Animal Research Committee of the Asan Institute for Life Sciences (IRB approval No. 2016–02-176).

**Fig. 1:**
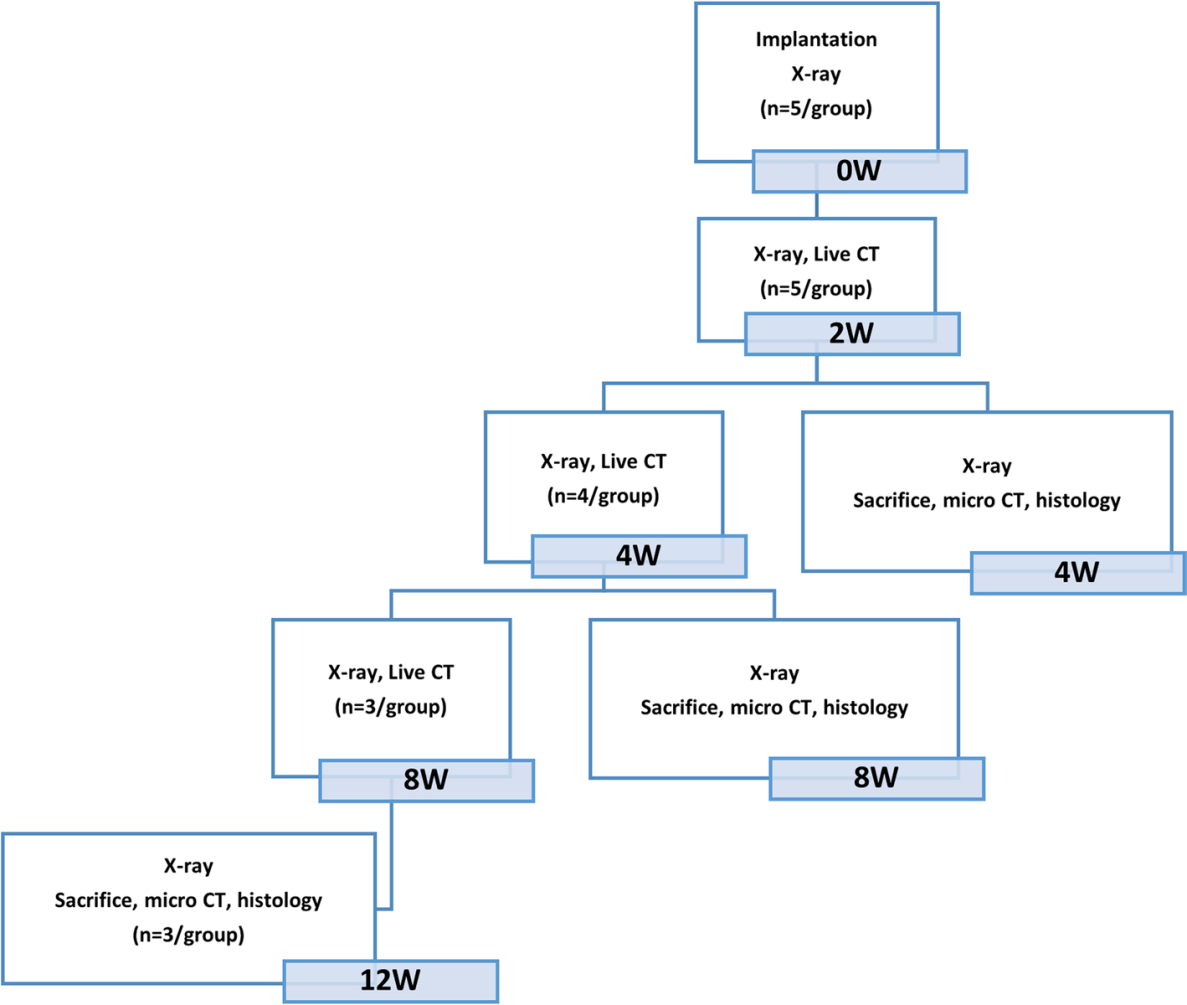
Flow chart of the present study

**Fig. 2:**
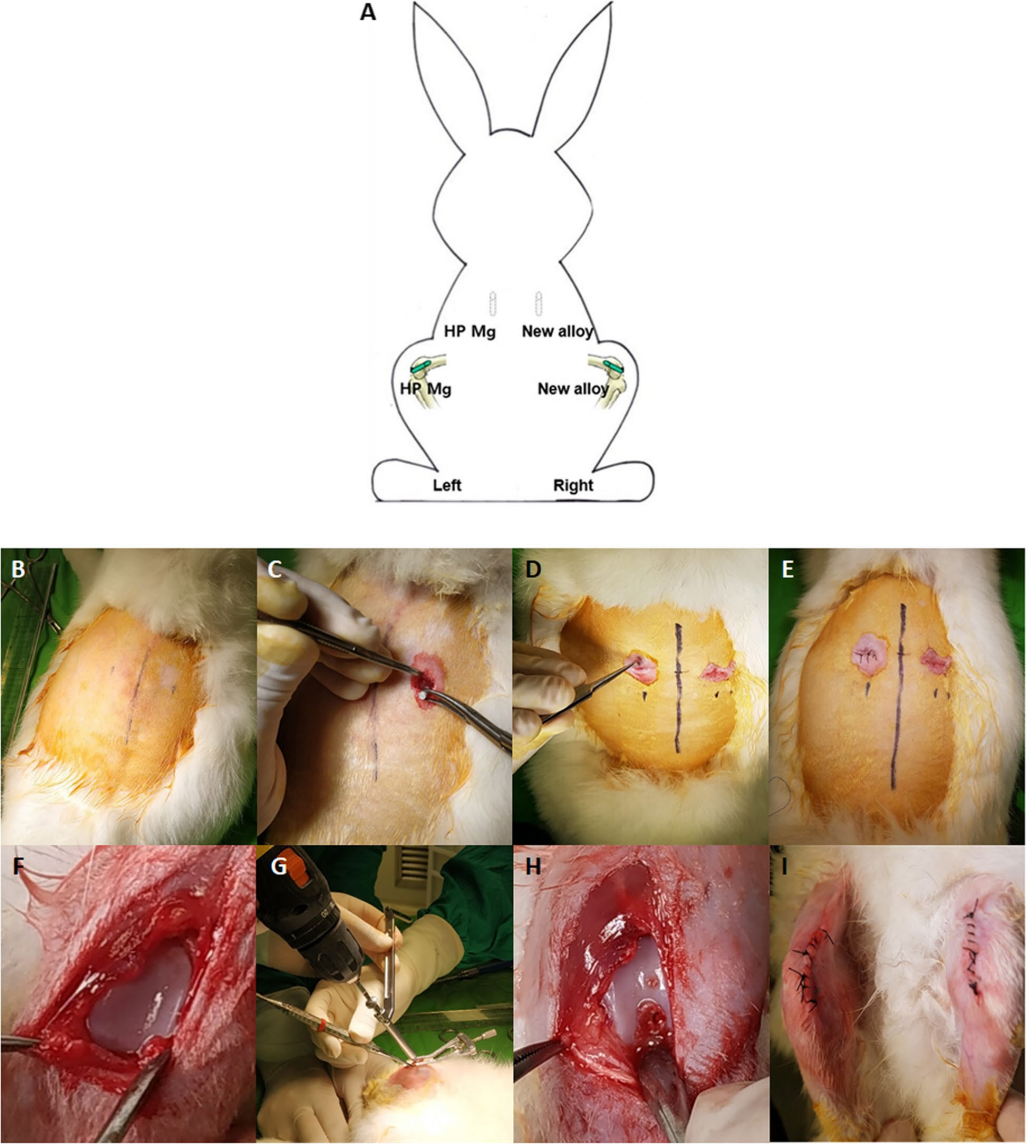
Implantation of the magnesium alloy devices into rabbit prevertebral muscles and femur condyle notches. **A**. Schematic of the implantation area. **B**. Hair was removed from the rabbit surgical site and the area was disinfected with povidone after administering general anesthesia. **C** and **D**. Each specimen was placed 2 cm in the left- and right-hand-sides of the muscle. **E**. The muscle was sutured to prevent separation of the specimen and the skin was sutured. **F**. The femur condyle notch was exposed. **G**. The site was tapped with a tapper after drilling with a drill guide. **H**. A screw was inserted with a cannulated screwdriver. I. Suturing was performed to complete the operation. HP, high purity; new alloy, Mg-1wt%Zn-0.1wt%Ca alloy with and without plasma electrolytic oxidation treatment

Nineteen New Zealand white rabbits were used in the study. Rod-bars (Ø 2.7 × 13.6 mm) were placed in both paravertebral muscles, and cannulated screws (Ø 2.7 × 10 mm) were attached to both femur condyle notches. The left and right sides were operated on in the same group and each animal was implanted in all four sites. To minimize the bias according to the location, the left/right implantation materials within the individual were alternately placed in the groups. X-ray images were taken at 0, 2, 4, 8, and 12 weeks, while micro-CT and live-CT scans were taken at 4, 8, and 12 weeks. At weeks 4, 8, and 12, individuals representing each group were selected and sacrificed to prepare specimens for histopathological examination (Fig. 1). Information on the implant materials according to the experimental animals and groups are reported in Table 1 and Fig. 2A.

**Table 1:** Experimental information of the study

**Animal species**	
New Zealand rabbit (male, 2.5 kg)	
**Animal age**	
12 weeks	
**Implant site (implant information)**	
Bilateral femur condyle notch (Ø 2.7 × 10 mm cannulated screw)	
Bilateral paravertebral muscle (Ø 2.7 × 13.6 mm rod bar)	
**Follow-up period**	
12 weeks	
**Groups**	**Materials**
Control	HP Mg (99.99 wt%)
Test 1	Mg-1wt%Zn-0.1wt%Ca alloy
Test 2	PEO-treated Mg-1wt%Zn-0.1wt%Ca alloy

*PEO* Plasma electrolytic oxidation

### Surgical procedures

#### Placement of the implant materials in the paravertebral muscle

Povidone was applied to the skin after removing the hair near the vertebral body where the implant material was to be placed. With the vertebral body as a reference, an incision of the minimum length to fit the specimen was made in the adjacent area 3 cm away from the vertebral body to the left and right. After the skin incision, the muscle was incised to secure a space so that the specimen could sufficiently fit inside and be surrounded by the muscle. The implant material was inserted into the muscle and incised muscle sutured to prevent the implant material from protruding during movement. After that, the skin was sutured in a routine manner to finish the procedure, the reason being to prevent overlapping caused by gas generation in both parts (Fig. 2B-E).

#### Placement of the implant materials in the femur condyle notch

Povidone was applied to the skin after removing the hair on the femur where the implant material was to be placed. After changing the animal’s posture to dorsal recumbency, the skin was incised with the right femur and tibia flexed in a fixed state, after which the muscle was carefully incised. The incision was stopped when the femur condyle notch became visible, after which drilling was attempted on the condyle with an electric drill (the drill bit was around 0.2 mm smaller than the diameter of the implant material). When drilling, a drill guide device was used to create a drilling hole suitable for the screw depth. The hole diameter was expanded so that the screw could be inserted by tapping the drilling hole space using a tapper. The screw was inserted into the drilling hole using a screwdriver, and after installation, the screw protrusion was placed so that the screw head was flat with the condyle surface to ensure that the screw head did not protrude above it. The procedure was completed by suturing the muscles and skin sequentially in a routine manner. The implant material was placed in the bone in the same way on the left side (Fig. 2F-I).

For each implant material, two animals from the test groups were placed on the right (or left) and two from the control groups on the left (or right) to check whether a total of 4 sets of implant materials had been inserted. In consideration of the bias according to the placement position, 5 out of 10 animals in each group were placed by changing the left and right positions of the test group and the control implant materials.

#### Management after surgery

After surgery, analgesics and anti-inflammatory drugs were administered once a day for a week, and abnormalities were determined through palpation of the surgical site, which was disinfected with povidone. When abnormal findings such as inflammation, hematoma, or skin swelling due to gas evolution were found at the implantation site, surgical staff would determine whether the surgical site was abnormal after imaging of the abnormal area and performing radiography with a portable X-ray device.

### Radiographical evaluation

#### X-ray scanning and analysis

X-ray (Comed, Gyunggi, Korea) images were taken immediately after surgery to check whether the placement was successful. X-ray scanner was performed with 60kVp, 200 mA, 0.06 sec, 12mAs. All of the animals were X-rayed at weeks 2, 4, 8, and 12 and before sacrificing.

#### Live-CT scanning and analysis

At weeks 2, 4, and 8, live-CT scanning (Somatom go.now, Munich, Germany) was conducted and gas evolution patterns over time were attained from the images, except for one animal per group that was sacrificed. Live-CT was performed with thickness 0.6 mm, reconstruction 0.5 mm(Bone window: Kernel value Hr60, SAFIRE 4, Soft tissue window: Kernel value Br40).

#### Micro-CT scanning and analysis

Micro-CT scanning was conducted after sacrificing one animal per group at weeks 4 and 8, and three animals per group at week 12. Bone and muscle samples were extracted and photographed with a SKYSCAN1172 (Bruker, Belgium) Micro-CT device at Asan Hospital, Seoul. The X-rays were transmitted at 50 kV, 200uA, and an Al 0.5 mm filter was used to capture the images with a resolution of 26.99 μm per pixel. An STL file was generated for the 3D modeling using a CT-Analyzer (Bruker, Kontich, Belgium), and the modeling was performed using the Ctvol (Bruker, Kontich, Belgium) program. The residual volume (mm^3^) was first measured in the acquired image, and then the in-vivo degradation rate (= initial volume-residual volume /initial surface/year) was calculated. For screws implanted into bone, bone-to-implant contact (BIC: %) [40, 41] was measured, which represents the ratio of the surface of the screw that remains in contact with the screw interface after corrosion.

**Fig. 3:**
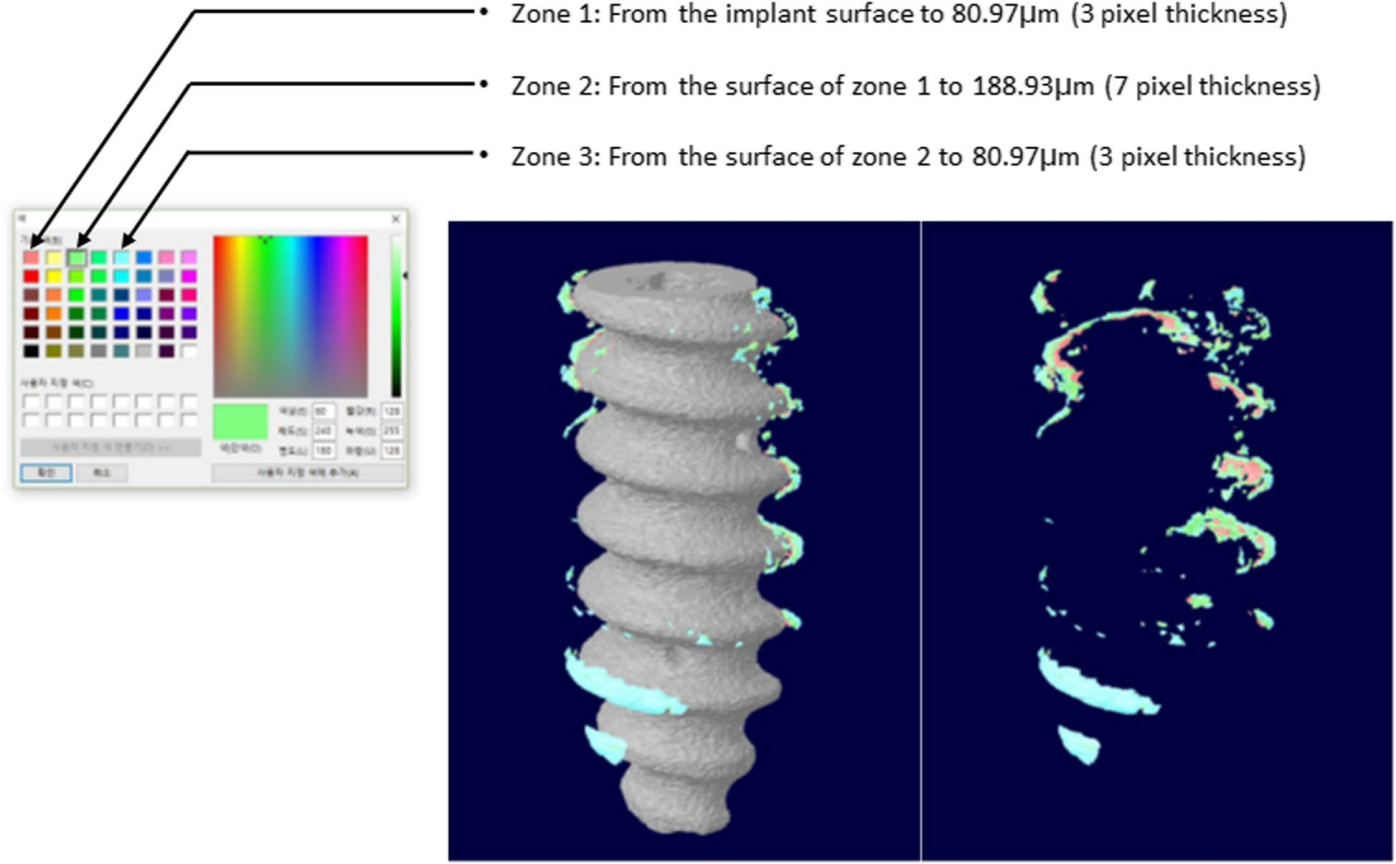
Bone implant contact (BIC) measurement section selection

Analysis of 33 rod-bars placed in the muscle and 33 bone screws 10 of each from the high-purity Mg group and 13 of each from the Mg-1wt%Zn-0.1wt%Ca alloy and PEO-treated Mg-1wt%Zn-0.1wt%Ca alloy groups) was conducted. The interfacial area of the screw implanted in the trabecular bone excluding the cortical bone at the screw placement site was selected to measure BIC. The measurement section was set at 80.97 μm from the screw interface (Fig. 3, Zone 1). The measurements of bone, tissue, and air the volumes within the section were converted to percentages for normalization. Among these values, the proportion of bone occupied was selected as BIC.

#### Histopathological evaluation

For the screw implanted in the bone, a slide that had been stained with Villanueva Osteochrome bone stain (BioLead Inc.) was observed to check the state of the screw and surrounding bone tissue. Muscle samples containing the implanted cylindrical rods were removed and fixed in formalin. Before performing Hematoxylin and Eosin staining, the graft material was removed from the muscle and stained (Seoul Asan medical center, Pathology Department). X12.5 slides were scanned at the test material implantation site to measure the above items. For the analysis, each item was evaluated by constantly expanding all areas at a high magnification of X400 based on the test substance interface.

#### Bone evaluation method

The histological evaluation method was conducted as described in [42] (Table 2). In addition, the degree of degradation of the screw, including cells that reflect bone remodeling around the screw implanted in the bone, the empty space in the part where the screw was degraded, and indicators of the decomposition residue were also evaluated.

**Table 2:** Histological evaluation system: cell type/response

Cell Type/Response	Score				
	0	1	2	3	4
Polymorphonuclear cells	0	Rare, 1–5/phf^a^	5–10/phf	Heavy infiltrate	Packed
Lymphocytes	0	Rare, 1–5/phf	5–10/phf	Heavy infiltrate	Packed
Plasma cells	0	Rare, 1–5/phf	5–10/phf	Heavy infiltrate	Packed
Macrophages	0	Rare, 1–5/phf	5–10/phf	Heavy infiltrate	Packed
Giant cells	0	Rare, 1–5/phf	5–10/phf	Heavy infiltrate	Packed
Necrosis	0	Minimal	Mild	Moderate	Severe
Neovascularisation	0	Minimal capillary proliferation, focal, 1–3 buds	Groups of 4–7 capillaries with supporting fibroblastic structures	Broad band of capillaries with supporting structures	Extensive band of capillaries with supporting fibroblastic structures
Fibrosis	0	Narrow band	Moderately thick band	Thick band	Extensive band
Fatty infiltrate	0	Minimal amount of fat associated with fibrosis	Several layers of fat and fibrosis	Elongated and broad accumulation of fat cells about the implant site	Extensive fat completely surrounding the implant

^a^ phf = per high powered (400 ×) field

#### Histomorphometric evaluation

**Fig. 4:**
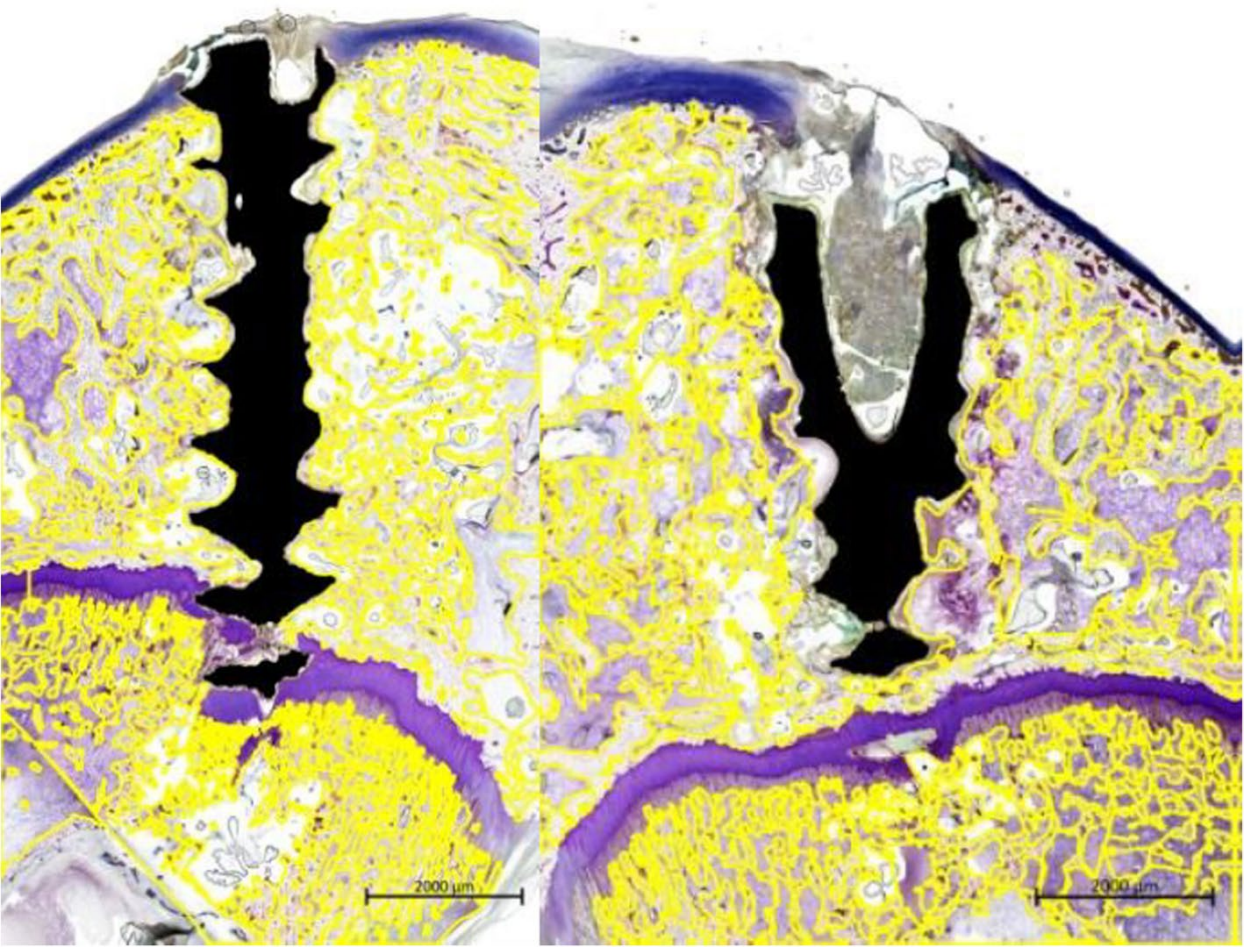
Region of interest designation to calculate the area of each evaluation item. Yellow fluorescent color denotes the regions of interest: the bone area, the implant area, the soft tissue area, and the void area within the total defect area, as well as the bone marrow area

Slides of bone were stained with Villanueva Osteochrome bone stain for morphometric and histopathological evaluation. Images were obtained using a slide scanner (Axio scan Z1, Carl Zeiss, Germany) to analyze the structure and shape of the bone tissue around the implant material. The following items were measured using an image analyzer (Zen 2.3 blue edition, Carl Zeiss, Germany): total defect area, bone area, implant area, soft tissue area, void area, and bone marrow area by scanning × 10 slides from the test material implantation sites. The images of the implant were analyzed using the manual and automatic ROI (region of interest) functions (Fig. 4). For the analysis of the measurement items, the area (μm^2^) was measured by selecting an ROI at 3 mm intervals based on the test material interface, including the bone area, the implant area, the soft tissue area, and the void area within the total defect area, as well as the bone marrow area. For normalization, the percentages of the area of ​​each item in the total defect area (100%) were calculated.

### Statistic

Differences between the groups were evaluated using Turkey’s multiple comparison test. All of the data are presented as the mean and standard deviation, and differences were considered to be statistically significant when *p* < 0.05. Statistical analyses were performed using SPSS software, version 27.0 J (SPSS, Inc., Chicago, IL, USA).

## Results

### Radiographical evaluation

**Fig. 5:**
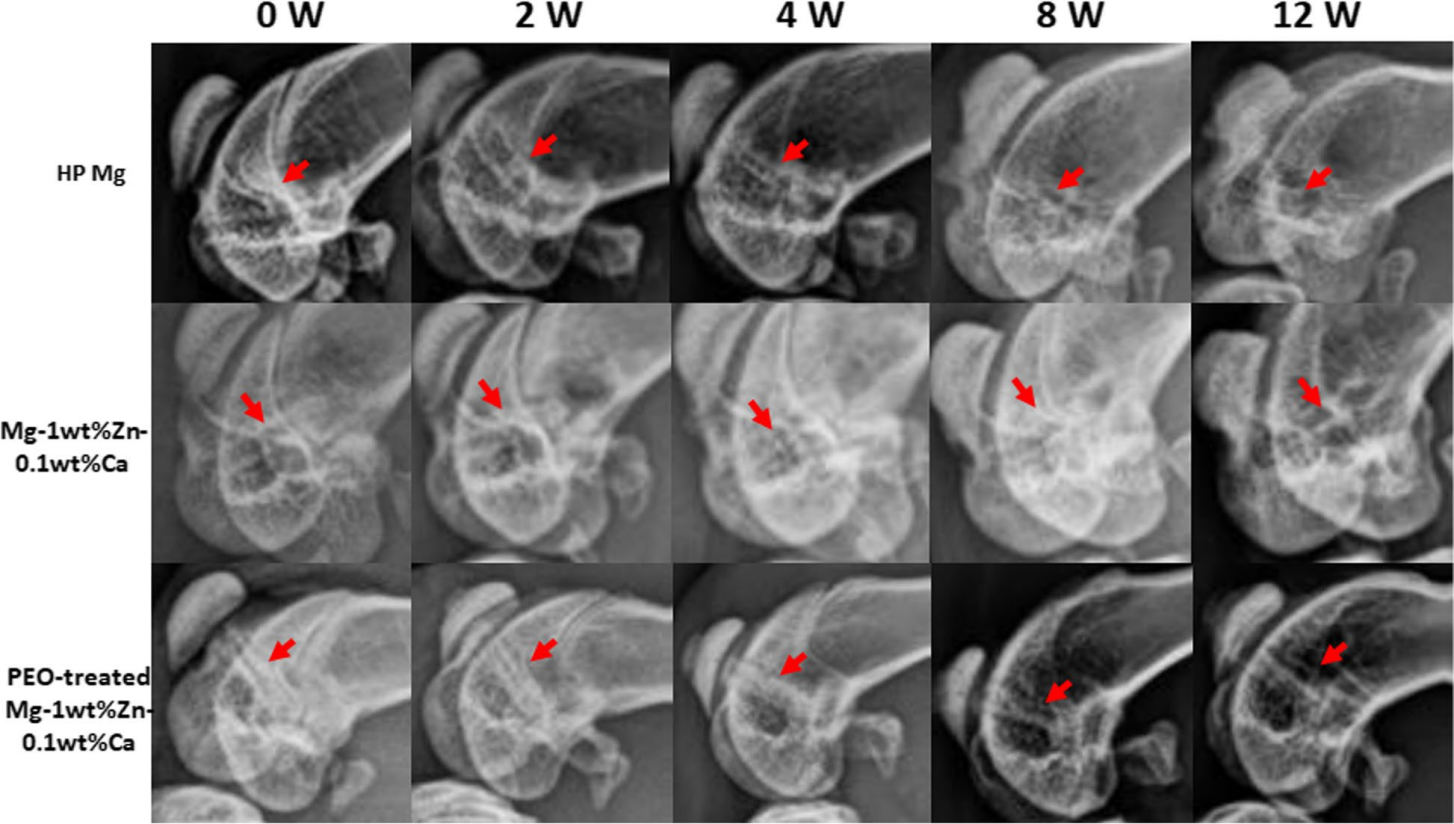
X-ray scan images of the rabbit femur condyle notch. X-rays were taken immediately after surgery and at 2, 4, 8, and 12 weeks after surgery. It can be observed that the graft material is well present in the surgical site over time. PEO, plasma electrolytic oxidation

**Fig. 6:**
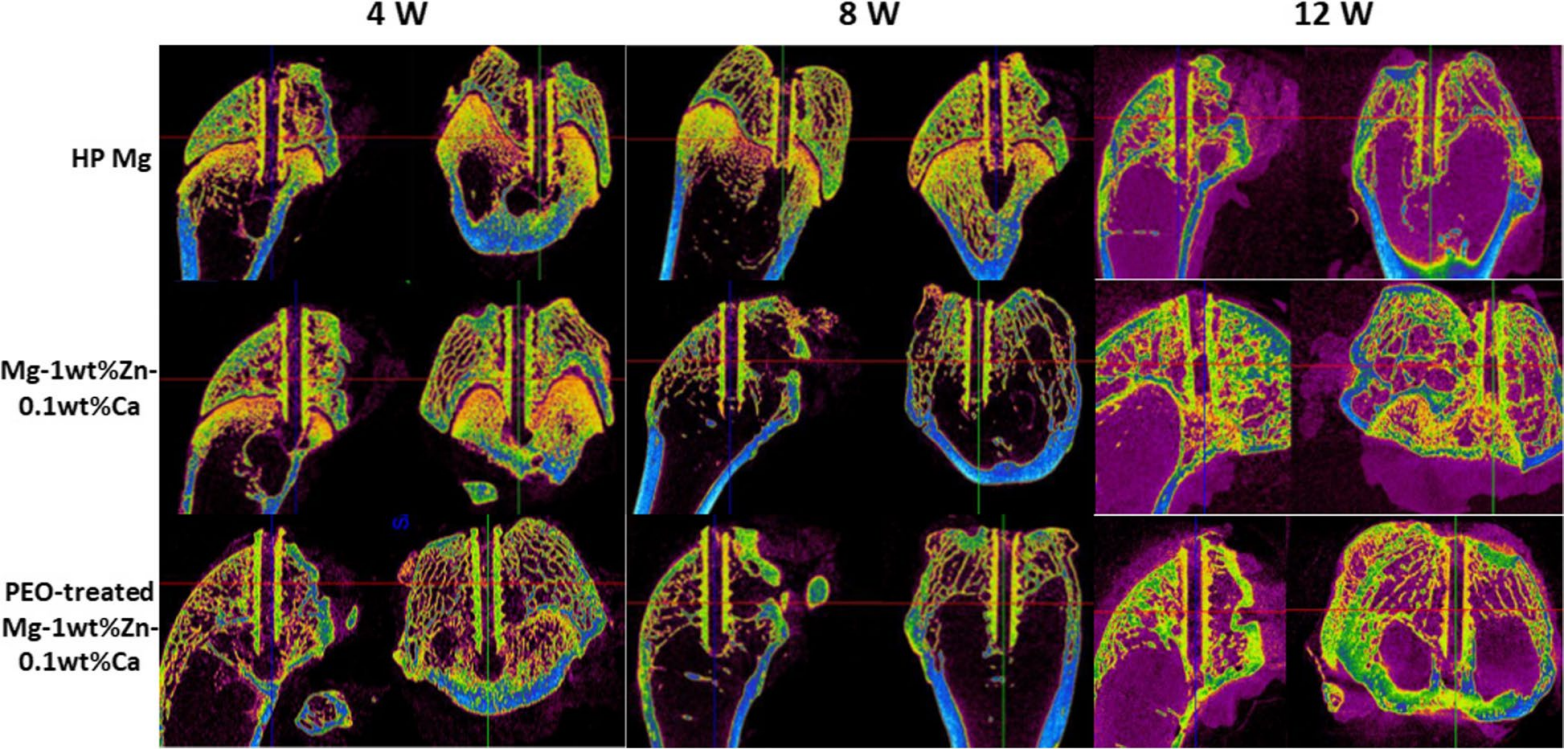
Micro-CT scan images of the rabbit femur condyle notch. Micro-CT scans were taken 4, 8, and 12 weeks after surgery and used to measure the implant volume, void volume, and degenerated bone volume. PEO, plasma electrolytic oxidation

**Fig. 7:**
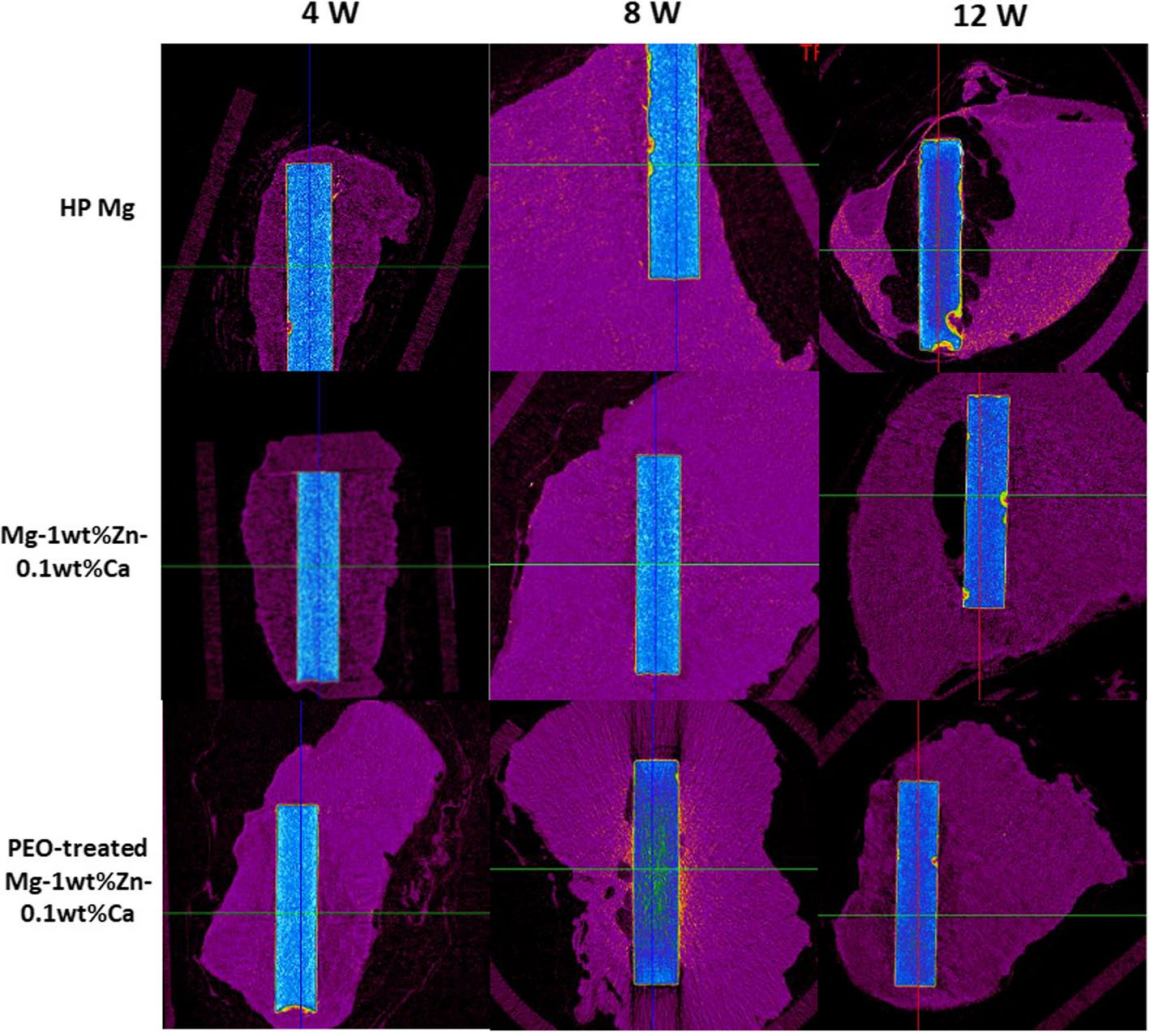
Micro-CT scan images of the rabbit prevertebral muscle. Micro-CT scans were taken 4, 8, and 12 weeks after surgery. The implant volume was measured using micro-CT data. PEO, plasma electrolytic oxidation

Figure 5 shows X-ray micrographs of the femur condyle notch of a rabbit with screws implanted for each planned week. Those taken immediately after the operations show that they proceeded without incident. Figures 6 and 7 present 2D micro-CT cross-sectional images at 4, 8, and 12 weeks after the implant insertions into the femur condyle notch and prevertebral muscle. In the analysis, the Mg alloy screw and the bone have similar average density, so there was no difference in contrast in the micro-CT images, and thus the boundary between the bone and the Mg screw was manually drawn once per 3 to 5 sections. The ROIs were manually specified and around 700 cross-sectional images per sample were taken. For all animals analyzed with data obtained after micro-CT tacking, the gas (empty space; black) generated by the screw at the implantation site was less than the previously reported trend [22], thus implying that the effect of the reduction of bone density due to gas buildup was smaller.

**Fig. 8:**
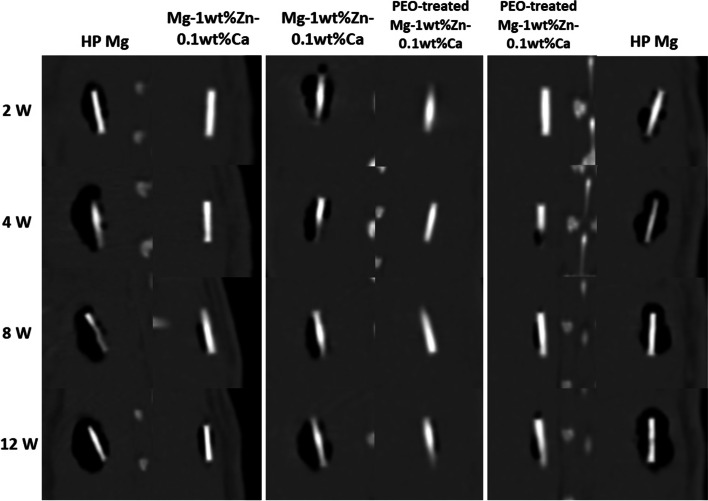
Live CT scan images of the rabbit prevertebral muscle. Live CT scans were taken 2, 4, 8, and 12 weeks after surgery. The amount of hydrogen gas increased from 2 weeks to 8 weeks post-implantation. The largest amount of hydrogen was produced by the high-purity Mg implant and the smallest by the plasma electrolytic oxidation (PEO)-treated Mg-1wt%Zn-0.1wt%Ca alloy

Figure 8 shows live-CT scans of the rabbit prevertebral muscle taken at 2, 4, 8, and 12 weeks after surgery, in which it can be seen that the amount of cavity by diffused hydrogen gas increased from 2 weeks to 8 weeks. When qualitatively comparing the amount of hydrogen generated by high-purity Mg, Mg-1wt%Zn-0.1wt%Ca alloy, and PEO-treated Mg-1wt%Zn-0.1wt%Ca alloy, the largest amount was by high-purity Mg and the smallest amount was by PEO-treated Mg-1wt%Zn-0.1wt%Ca alloy.

**Fig. 9:**
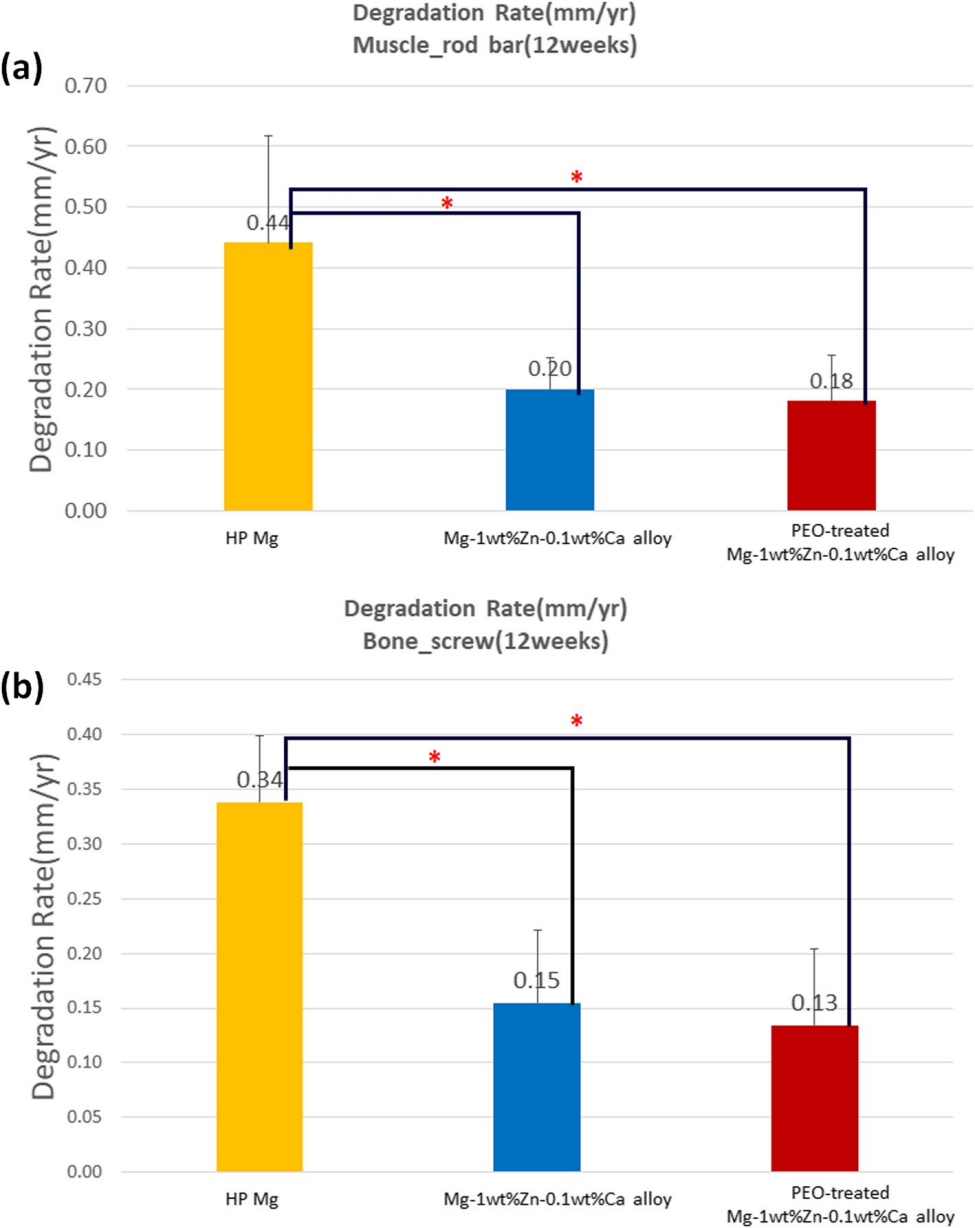
3D modeling of screws and degradation rate. **a** Degradation rates of the screws implanted in bone. **b** Degradation rates of the screws implanted in muscle. HP, high-purity; PEO, plasma electrolytic oxidation (*, *p* < 0.05)

Figure 9 (a), (b) show the in-vivo degradation rates calculated using residual volume values. From the results, the degradation rate of the Mg-1wt%Zn-0.1wt%Ca alloy implants was slower than that of the control high-purity Mg ones, while the PEO-treated Mg-1wt%Zn-0.1wt%Ca alloy implants seem to be degraded slowly.

**Fig. 10:**
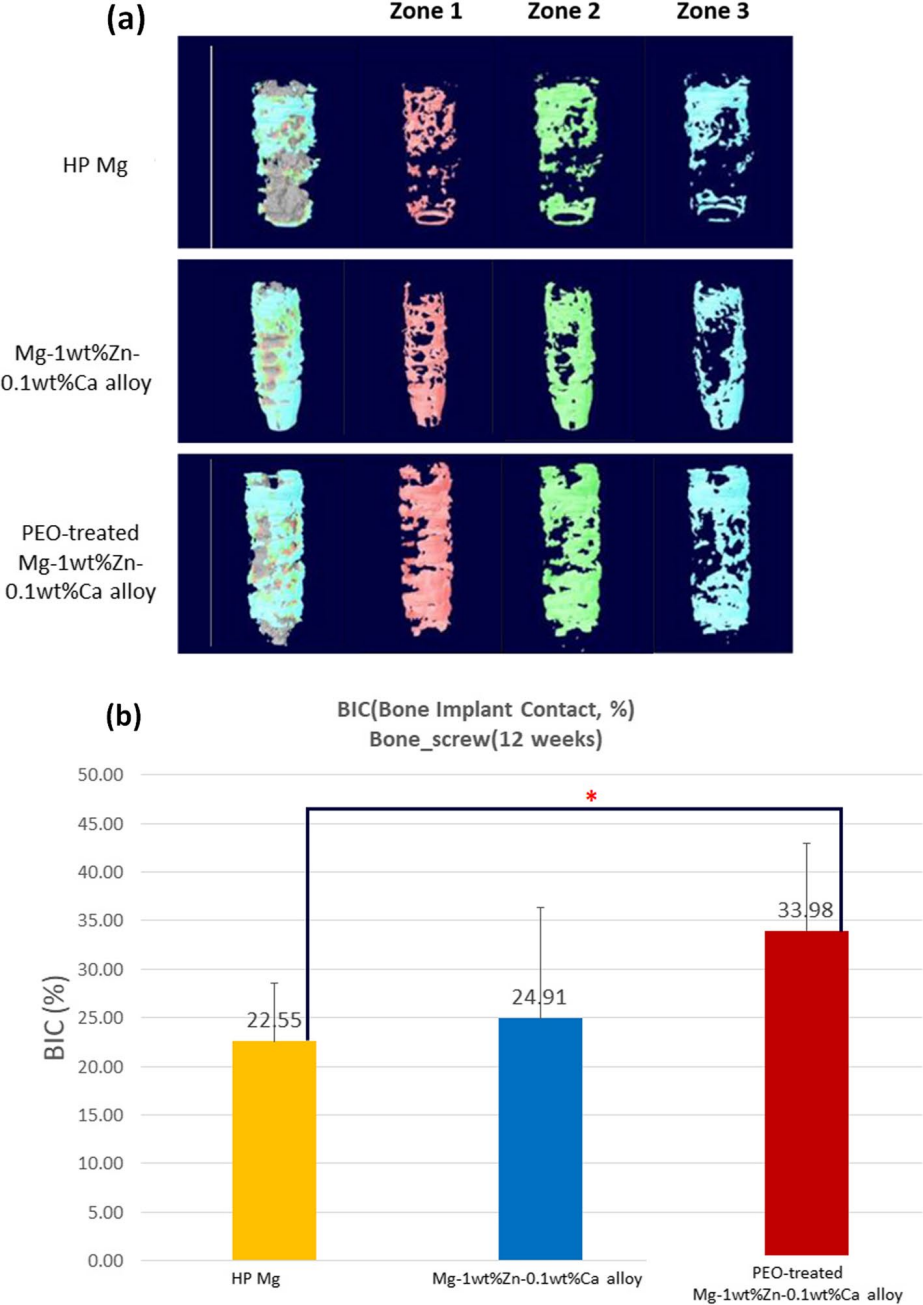
BIC (bone implant contact) 3D modeling and measurements. **a** BIC 3D modeling of screws placed in rabbit femur condyle notches at 12 weeks. **b** BIC measurements of screws placed in rabbit femur condyle notches. HP, high-purity; PEO, plasma electrolytic oxidation (*, *p* < 0.05)

Figure 10 (a) presents the 3D modeling of each implanted material to measure BIC. Based on the distance from the screw surface, zones 1 to 3 (Fig. 3) were divided and the bone occupied ratio was calculated. The BIC values were in the order of PEO-treated Mg-1wt%Zn-0.1wt%Ca alloy > Mg-1wt%Zn-0.1wt%Ca alloy > high-purity Mg (Fig. 10(b)).

### Histopathological evaluation

#### Histopathologic results from un-decalcified bone slides

**Fig. 11:**
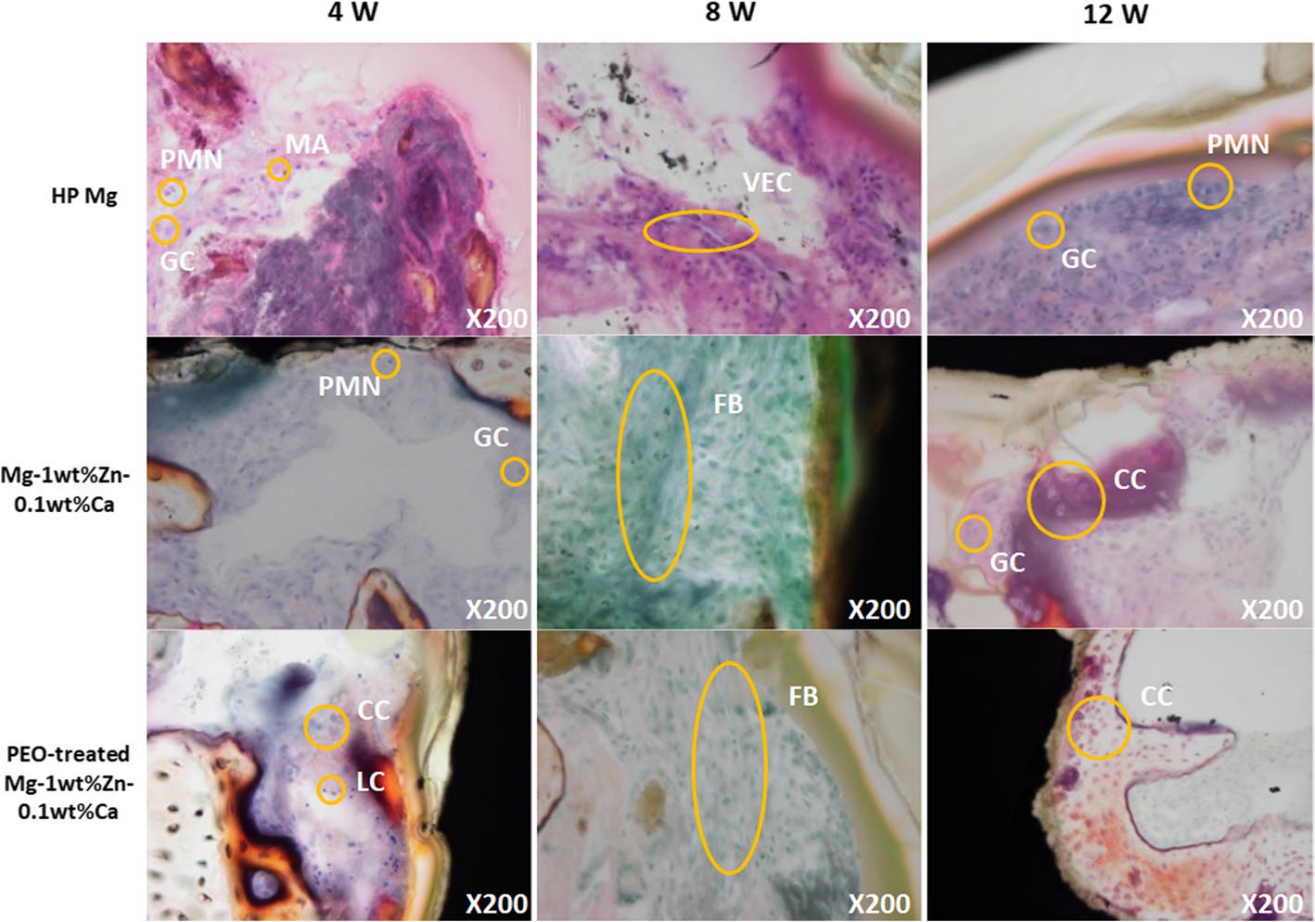
Villanueva Osteochrome bone staining of a rabbit femur condyle notch 4, 8, 12 weeks after implantation. PMN, polymorphonuclear cell; MA, macrophage; GC, Giant cell; CC, chondrocyte; LC, lymphocyte; VEC, vascular endothelial cell; FB, fibroblast

Bone tissue was observed in the implant interface of the test groups because the cartilage of the joint or growth plate was released during the procedure. The invasion level of inflammatory cells such as polymorphonuclear cells, lymphocytes, macrophages, and giant cells in bone tissue was observed at similar levels in all tested subjects in the 4th and 8th week specimens (Fig. 11). For the 12-week specimens, the infiltration of inflammatory cells decreased in all groups, which was more pronounced in the test groups than the control group. In particular, the PEO-treated Mg-1wt%Zn-0.1wt%Ca alloy group showed almost no inflammatory response (Fig. 11). At week 12 (when bone union was completed), the PEO-treated Mg-1wt%Zn-0.1wt%Ca alloy group showed little inflammatory response compared to the control group, indicating that the PEO-coated Mg-1wt%Zn-0.1wt%Ca alloy implants were the least irritating (Table 3).

**Table 3:** Histopathological examination of un-decalcified bone slide (Villanueva Osteochrome bone stain)

Sample			Polymorphonuclear cell	Lymphocytes	Plasma Cells	Macrophages	Giant Cells	Necrosis	Sub-Total (×2)	Neovascularization	Fibrosis	Fatty Infiltrate	Sub-Total	Total
1	Control	4w	0	2	–	2	1	0	10	1	1	0	2	12
2	Test 1	4w	0	1	–	1	1	0	6	0	1	0	1	7
3	Test 2	4w	1	2	–	1	1	0	10	2	1	0	3	13
4	Control	8w	1	2	–	1	2	0	12	1	2	0	3	15
5	Test 1	8w	1	1	–	1	1	0	8	2	2	0	4	12
6	Test 2	8w	1	1	–	2	1	0	10	2	2	0	4	14
7	Control	12w	1	1	–	2	1	0	8	2	1	0	3	11
8	Control	12w	1	0	–	1	1	0	5	1	2	0	3	8
9	Test 1	12w	0	0	–	0	0	0	0	0	0	0	0	0
10	Test 1	12w	0	0	–	1	1	0	4	2	1	0	3	7
11	Test 2	12w	0	0	–	0	0	0	0	0	0	0	0	0
12	Test 2	12w	0	0	–	1	0	0	2	1	2	0	3	4

(−) Unable to check due to a problem with the thickness of the produced tissue sample. (Control: HP Mg, Test 1: Mg-1wt%Zn-0.1wt%Ca alloy, Test 2: PEO-treated Mg-1wt%Zn-0.1wt%Ca alloy)

#### Histopathologic results for the muscle tissue

**Fig. 12:**
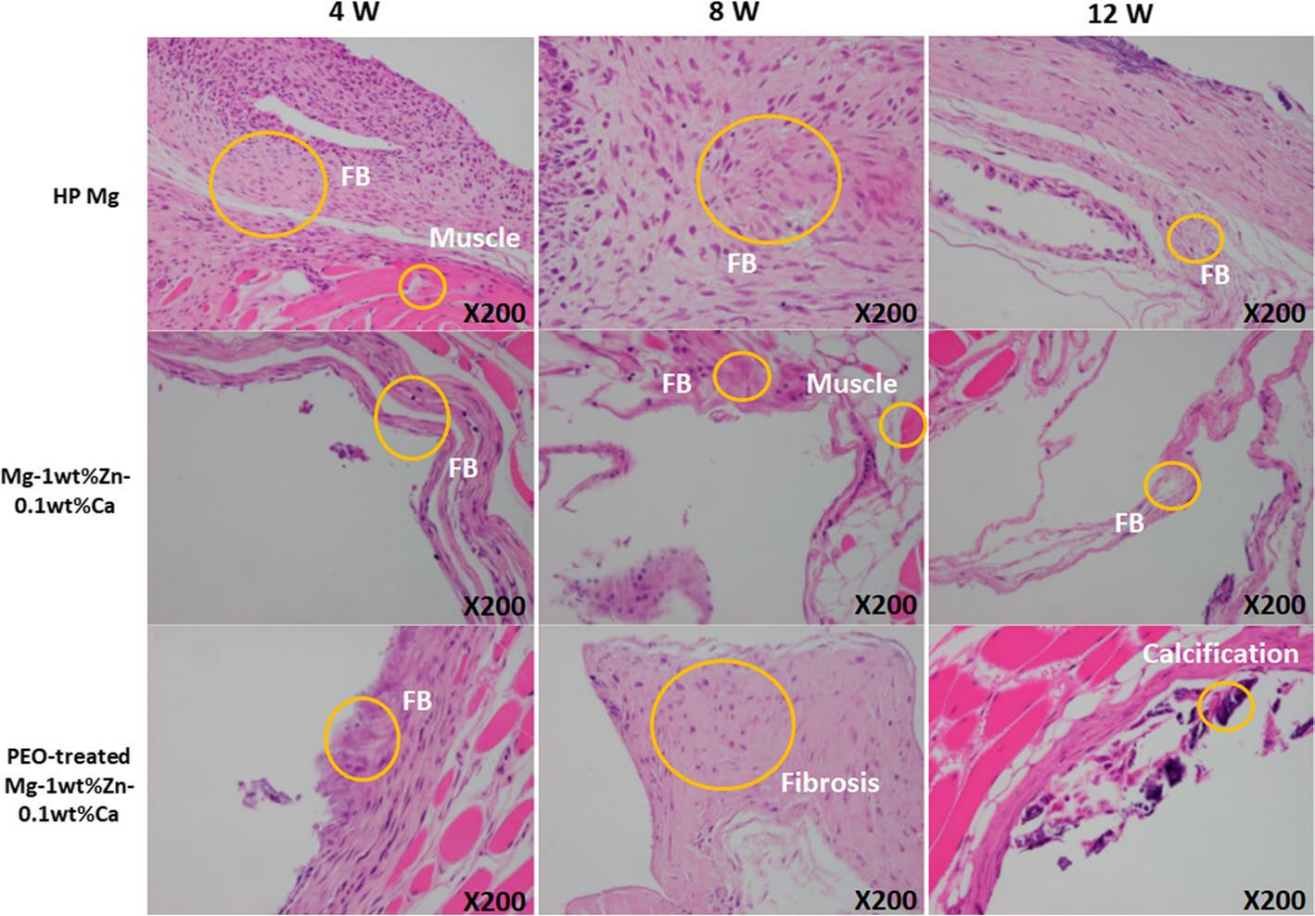
Hematoxylin and Eosin staining of a rabbit prevertebral muscle area 4, 8, 12 weeks after implantation. FB, fibroblast

Fibrosis due to muscle necrosis was observed in the control group at week 4 (Fig. 12), and dystrophic calcification was also observed in the fibrotic tissues in the Mg-1wt%Zn-0.1wt%Ca alloy and PEO-treated Mg-1wt%Zn-0.1wt%Ca alloy groups (Fig. 12) at weeks 8 and 12. In the histopathological examination, the total scores of the control group at weeks 4 and 8 were 20 and 29 points, respectively, with the latter being the highest among the groups (Table 4). The combined scores of the Mg-1wt%Zn-0.1wt%Ca alloy and PEO-treated Mg-1wt%Zn-0.1wt%Ca alloy test groups were 11 and 14 points at week 4 and 10 and 15 points at week 8, respectively, which was lower than that of the control group. At 12 weeks, the average values for the control group and Mg-1wt%Zn-0.1wt%Ca alloy groups and PEO-treated Mg-1wt%Zn-0.1wt%Ca alloy were 17, 11, and 8 points, once again indicating that the PEO-treated Mg-1wt%Zn-0.1wt%Ca alloy implants were the least irritating.

**Table 4:** Histopathological examination of muscle slides (Hematoxylin and Eosin stain)

Sample			Polymorphonuclear Cells	Lymphocytes	Plasma Cells	Macrophages	Giant Cells	Necrosis	Sub-Total (×2)	Neovascularization	Fibrosis	Fatty Infiltrate	Sub-Total	Total
1	Control	4w	1	1	0	4	1	0	14	2	3	1	6	20
2	Test 1	4w	0	1	1	0	1	0	6	2	2	1	5	11
3	Test 2	4w	1	0	0	1	1	1	8	1	2	3	6	14
4	Control	8w	1	1	0	4	1	4	22	2	4	1	7	29
5	Test 1	8w	0	1	0	2	0	0	6	1	1	2	4	10
6	Test 2 l	8w	1	0	0	1	1	1	8	1	3	3	7	15
7	Control	12w	0	1	0	1	0	1	6	3	1	3	7	13
8	Control	12w	0	1	0	2	1	0	8	2	2	4	8	16
9	Control	12w	0	3	0	3	1	1	16	2	3	2	7	23
10	Test 1	12w	1	1	0	1	1	0	8	2	2	2	6	14
11	Test 1	12w	0	0	0	0	0	1	2	1	2	3	6	8
12	Test 2	12w	0	0	0	0	0	0	0	1	1	1	3	3
13	Test 2	12w	0	0	0	1	2	2	10	0	2	1	3	13
14	Test 2	12w	0	0	0	2	0	0	4	1	3	1	5	9

(Control: HP Mg, Test 1: Mg-1wt%Zn-0.1wt%Ca alloy, Test 2: PEO-treated Mg-1wt%Zn-0.1wt%Ca alloy)

#### Histomorphometric test results

**Fig. 13:**
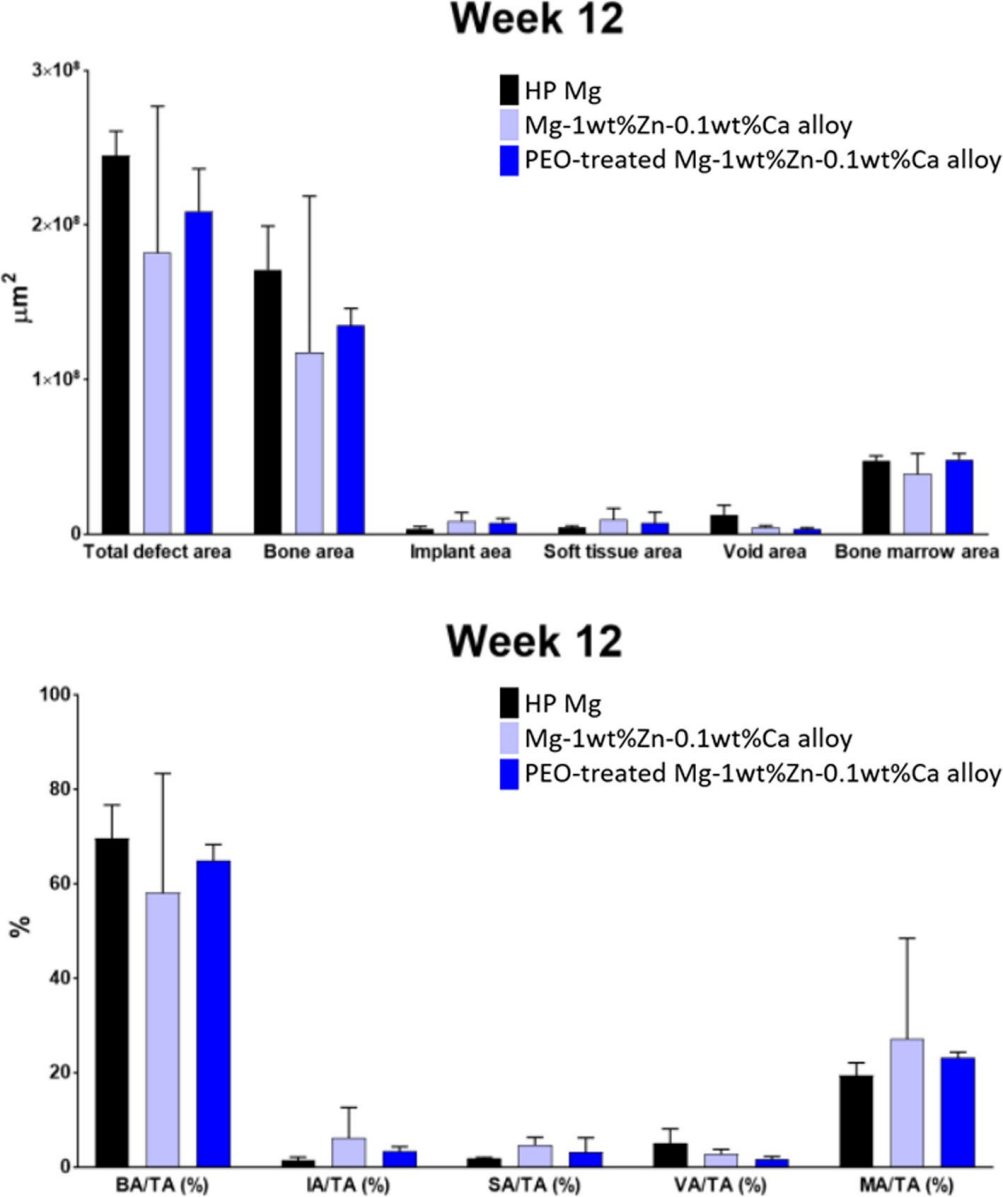
Histomorphometric examination results. TA, total defect area; BA, bone area; IA, implant area; SA, soft tissue area; VA, void area; BM, bone marrow area

In the 12th week specimens, the PEO-treated Mg-1wt%Zn-0.1wt%Ca alloy group showed a smaller defect area than that of the control group and the Mg-1wt%Zn-0.1wt%Ca alloy group. In the 12th week specimens, the bone area was the smallest in the Mg-1wt%Zn-0.1wt%Ca alloy group. The soft tissue area of each group at the 12th weeks showed similar levels. The void area of 12th weeks, control group and Mg-1wt%Zn-0.1wt%Ca alloy group were higher than the PEO-treated Mg-1wt%Zn-0.1wt%Ca alloy group, although the difference is not statistically significant (Fig. 13).

## Discussion

The aim of this study was to compare the biocompatibility of three biodegradable materials: high-purity Mg, Mg-1wt%Zn-0.1wt%Ca alloy, and PEO-treated Mg-1wt%Zn-0.1wt%Ca alloy. Implantation into a rabbit’s femur and muscle was selected as the animal model as it was frequently used as fundamental research method for testing biocompatibility of various magnesium alloys in the past fundamental research of biocompatibility of various magnesium alloys [43, 44].

Patellar dislocation occurred in 5 rabbits, as observed in the 2-week postoperative X-ray radiographs. Recent studies have proven that dislocation of the patella early in the rabbit’s development can lead to femoral trochlear dysplasia [45] and tibial tubercle lateralization [46]. In this study, patella dislocation occurred due to peripheral ligament damage during surgical intervention. Three additional rabbits were operated on to establish study consistency. Mild subcutaneous emphysema occurred at 2 and 4 weeks post implantation in the high-purity Mg group as well as at 8 weeks in the Mg-1wt%Zn-0.1wt%Ca alloy group. Since hydrogen is formed during the degradation of Mg [12, 20], it is not surprising to discover gas bubbles during the investigations of the respective implants. Some authors have concluded that hydrogen diffuses into the tissue and is thus only visible as gas bubbles during very rapid degradation [47].

The degradation rates of the rod-bars and screws were calculated using the residual volume values from Fig. 9 (a) and (b), respectively; the corrosion rate of Mg-1wt%Zn-0.1wt%Ca alloy was slower than that of pure Mg, and the PEO-treated Mg-1wt%Zn-0.1wt%Ca alloy showed slower figure. It is reported that a small amount of Zn less than 3% can increase the corrosion potential of magnesium alloys and improve corrosion resistance [48, 49], which is consistent with this study (Fig. 9 (a) and (b)). It has also been reported that Zn can increase the charge transfer resistance of Mg, thereby lowering the corrosion rate [50]. PEO treatment creates a protective oxide layer with a high degree of porosity, which delays the initial corrosion process and improves the formation of primary new bone around the implanted material, thereby resulting in reduced hydrogen evolution [51].

The interfacial region of the screw implanted in the trabecular bone excluding the cortical bone was selected to measure the BIC, which represents the ratio of the surface in contact with the bone to the screw interface. The volume of bone, tissue, and air occupied within the section was measured after determining the measurement section from Zone 1 to 3, and the proportion of bone was selected as BIC after converting the values to percentages for normalization. The results indicate that the higher the BIC, the higher the corrosion resistance, and the less hydrogen gas is generated. The BIC values were in the order of PEO-treated Mg-1wt%Zn-0.1wt%Ca alloy > Mg-1wt%Zn-0.1wt%Ca alloy > high-purity Mg.

From the results of the histopathological observations from un-decalcified bone slides, bone tissue was observed in the interface of the test groups due to the release of cartilage (Fig. 11) from the joint or growth plate during the procedure. Inflammatory cell infiltration was observed in polymorphonuclear cells, lymphocytes, macrophages, and giant cells in the bone tissue (Fig. 11). The inflammatory cells were probably due to foreign body reaction after implantation, and similar levels of inflammatory cell infiltration were observed in all 4- and 8-week autopsies. In the 12th week specimens, the invasion of inflammatory cells had decreased in all groups, which was most noticeable in the test groups (Table 3). In particular, the inflammatory response was very low in the PEO-treated Mg-1wt%Zn-0.1wt%Ca alloy group. At week 12 (when bone union was completed), this group showed little inflammatory response compared to the control group, thus the PEO-treated Mg-1wt%Zn-0.1wt%Ca alloy implants were the least irritating.

From the observations of lesion level in the muscle tissue, the level of inflammatory cell infiltration, along with the total scores for angiogenesis, fibrosis, and lipid bleeding, were lower in the 4th and 8th week for both test groups compared to the control group (Table 4). The overall inflammatory cell infiltration was reduced in all groups, with the values of the test groups being lower than that of the control group. Therefore, the Mg-1wt%Zn-0.1wt%Ca alloy and PEO-treated Mg-1wt%Zn-0.1wt%Ca alloy implants caused less irritation to muscle tissue than the control group. For normalization the percentages of the area of each item in the total defect area (100%) were calculated (Fig. 13). The histomorphometric examination results from the un-decalcified bone slides showed that the recovery pattern was slightly improved after tissue damage in the PEO-treated Mg-1wt%Zn-0.1wt%Ca alloy group, because the VA/TA (%) of the 12 weeks PEO-treated group was smaller than that of the other groups, even though there is no statistically significant difference (Fig. 13).

The main limitation of this study was that we only conducted a qualitative evaluation of cavities found in Live-CT. The H_2_ gas generated as magnesium decomposes diffused rapidly into the body, so the amount of gas generated could not be measured. Second, there were no significant differences in the BIC and degradation rates between the Mg-1wt%Zn-0.1wt%Ca alloy and the PEO-treated Mg-1wt%Zn-0.1wt%Ca alloy, although this limitation will be present in any animal experiment conducted with small n numbers.

In this study, the Mg-1wt%Zn-0.1wt%Ca alloy and PEO-treated Mg-1wt%Zn-0.1wt%Ca alloy groups showed similar levels of inflammation in the bone tissue observations compared to the control group in weeks 4 and 8. Moreover, the overall inflammation level decreased in the 12th week in all groups compared to the 4th and 8th weeks, with a faster recovery pattern being observed in both test groups compared to the control group. From the muscle tissue observations, both test groups showed lower inflammation levels at weeks 4 and 8 compared to the control group (Tables 3 and 4), and inflammatory cell infiltration at week 12 was reduced in all groups compared to weeks 4 and 8, with lower values ​​being observed in the test groups than the control group. Therefore, the Mg-1wt%Zn-0.1wt%Ca alloy and PEO-treated Mg-1wt%Zn-0.1wt%Ca alloy implants were less irritating to the muscle tissue than the high-purity Mg ones. The bone tissue morphological results for the groups were similar ​​at week 4, but the void areas at weeks 8 and 12 were significantly decreased in the PEO-treated Mg-1wt%Zn-0.1wt%Ca alloy group compared to the others (Fig. 13). Thus, it was confirmed that the PEO-treated Mg-1wt%Zn-0.1wt%Ca alloy implants produced less gas at the interface between the device and the tissue.

## Conclusions

In the present study we assessed the biocompatibility evaluation of PEO-treated magnesium alloy implants placed in rabbit femur condyle notches and paravertebral muscles. It was confirmed that Mg-1wt%Zn-0.1wt%Ca alloy had higher corrosion resistance than high-purity Mg and safely degraded over time without causing side effects (foreign body reaction, inflammatory reaction, etc.) in vivo. In addition, post-treatment of Mg-1wt%Zn-0.1wt%Ca alloy via PEO was found to have a positive effect on fracture recovery, such as increasing the bonding area with bone. Based on these results, PEO treatment of Mg-1wt%Zn-0.1wt%Ca alloy can be a promising biomaterials in the field of various clinical situations such as orthopedic and maxillofacial surgeries.

## Supplementary Information

**Additional file 1.**

**Additional file 2.**

## Data Availability

Not applicable.

## Abbreviations

BIC: Bone implant contact
HP: High purity
PEO: Plasma electrolytic oxidation
PMN: Polymorphonuclear cell
MA: Macrophage
GC: Giant cell
CC: Chondrocyte
LC: Lymphocyte
VEC: Vascular endothelial cell
FB: Fibroblast
TA: Total defect area
BA: Bone area
IA: Implant area
SA: Soft tissue area
VA: Void area
BM: Bone marrow area
