# Supplementary figures and images for: Biocompatibility evaluation of peo-treated magnesium alloy implants placed in rabbit femur condyle notches and paravertebral muscles
Source: Biomater Res. 2022 Jul 6;26:29. doi: 10.1186/s40824-022-00279-1 (PMC9258108; doi:10.1186/s40824-022-00279-1)

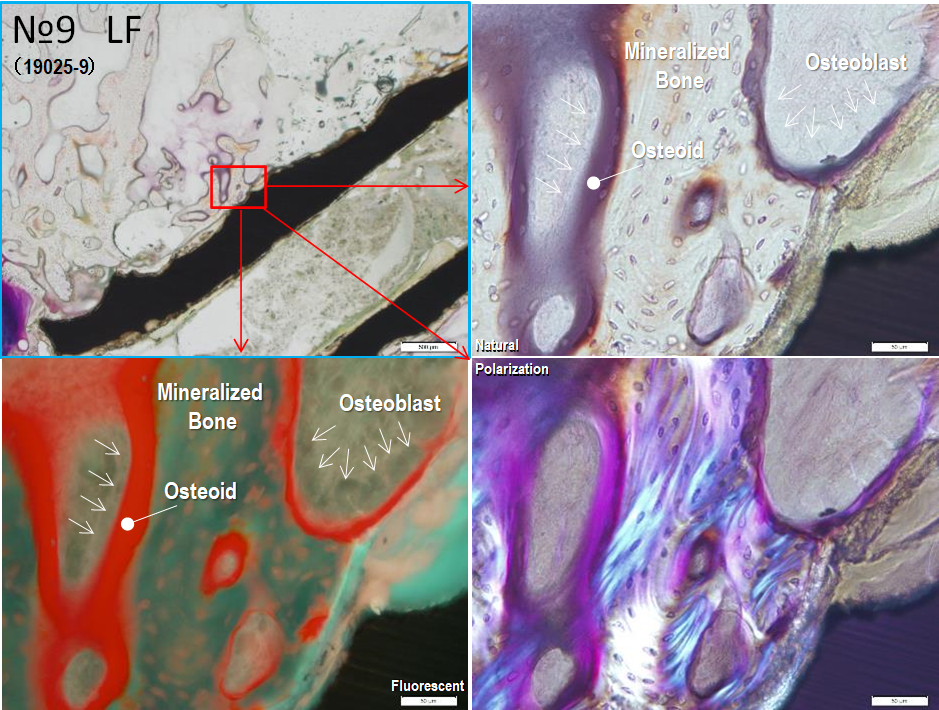

Supplement: Supplementary file 1 — Additional file 1. [file 40824_2022_279_MOESM1_ESM.tif]
