# Supplementary material for: Biocompatibility evaluation of peo-treated magnesium alloy implants placed in rabbit femur condyle notches and paravertebral muscles
Source: Biomater Res. 2022 Jul 6;26:29. doi: 10.1186/s40824-022-00279-1 (PMC9258108; doi:10.1186/s40824-022-00279-1)
Supplement: Supplementary file 2 — Additional file 2. [file 40824_2022_279_MOESM2_ESM.docx]

| **H2 gas generation rate(mL/㎠/hr)**  **Non-Coated(N=12)** | | | | | | | |  |  |  |  |  |  |  |  |  |  |  |  |  |  |  |  |  |  |  |  |  |  |  |  |  |  |  |  |  |  |  |  |  |  |  |  |
| --- | --- | --- | --- | --- | --- | --- | --- | --- | --- | --- | --- | --- | --- | --- | --- | --- | --- | --- | --- | --- | --- | --- | --- | --- | --- | --- | --- | --- | --- | --- | --- | --- | --- | --- | --- | --- | --- | --- | --- | --- | --- | --- | --- |
|  | 0.5 | 1 | 2 | 4 | 8 | 24 | 48 | 72 | 96 | 120 | 144 | **168** | 240 | 264 | 288 | 312 | 336 | 408 | 432 | 456 | 480 | **504** | 576 | 600 | 648 | 672 | 744 | 792 | **840** | 912 | 984 | **1104** | 1128 | 1152 | 1176 | 1296 | 1320 | 1440 | 1488 | 1632 | 1752 | 1776 | 1800 |
| 1 | 0.000 | 0.018 | 0.009 | 0.009 | 0.009 | 0.007 | 0.006 | 0.006 | 0.006 | 0.005 | 0.004 | 0.004 | 0.004 | 0.004 | 0.003 | 0.003 | 0.003 | 0.004 | 0.003 | 0.004 | 0.004 | 0.004 | 0.004 | 0.004 | 0.004 | 0.004 | 0.003 | 0.003 | 0.003 | 0.003 | 0.003 | 0.003 | 0.003 | 0.003 | 0.003 | 0.002 | 0.002 | 0.002 | 0.002 | 0.002 | 0.002 | 0.002 | 0.002 |
| 2 | 0.000 | 0.000 | 0.009 | 0.009 | 0.009 | 0.013 | 0.011 | 0.011 | 0.010 | 0.009 | 0.008 | 0.008 | 0.013 | 0.013 | 0.012 | 0.011 | 0.011 | 0.011 | 0.011 | 0.011 | 0.010 | 0.010 | 0.010 | 0.010 | 0.009 | 0.009 | 0.009 | 0.009 | 0.008 | 0.008 | 0.008 | 0.007 | 0.007 | 0.007 | 0.007 | 0.006 | 0.006 | 0.006 | 0.005 | 0.005 | 0.005 | 0.005 | 0.005 |
| 3 | 0.000 | 0.000 | 0.000 | 0.004 | 0.009 | 0.013 | 0.012 | 0.012 | 0.011 | 0.010 | 0.009 | 0.008 | 0.009 | 0.009 | 0.009 | 0.009 | 0.008 | 0.009 | 0.009 | 0.007 | 0.006 | 0.006 | 0.006 | 0.006 | 0.005 | 0.005 | 0.005 | 0.005 | 0.005 | 0.005 | 0.005 | 0.005 | 0.005 | 0.005 | 0.005 | 0.005 | 0.004 | 0.004 | 0.004 | 0.004 | 0.004 | 0.004 | 0.004 |
| 4 | 0.000 | 0.000 | 0.009 | 0.009 | 0.011 | 0.013 | 0.011 | 0.011 | 0.010 | 0.009 | 0.008 | 0.008 | 0.008 | 0.007 | 0.007 | 0.007 | 0.007 | 0.007 | 0.007 | 0.007 | 0.007 | 0.007 | 0.008 | 0.008 | 0.007 | 0.007 | 0.007 | 0.007 | 0.007 | 0.007 | 0.006 | 0.006 | 0.006 | 0.006 | 0.006 | 0.005 | 0.005 | 0.005 | 0.005 | 0.004 | 0.004 | 0.004 | 0.004 |
| 5 | 0.000 | 0.018 | 0.018 | 0.013 | 0.013 | 0.011 | 0.011 | 0.010 | 0.009 | 0.008 | 0.007 | 0.007 | 0.006 | 0.006 | 0.005 | 0.005 | 0.005 | 0.005 | 0.005 | 0.004 | 0.004 | 0.004 | 0.004 | 0.004 | 0.004 | 0.004 | 0.004 | 0.004 | 0.004 | 0.004 | 0.003 | 0.003 | 0.003 | 0.003 | 0.003 | 0.003 | 0.003 | 0.002 | 0.002 | 0.002 | 0.002 | 0.002 | 0.002 |
| 6 | 0.000 | 0.000 | 0.009 | 0.009 | 0.011 | 0.010 | 0.007 | 0.006 | 0.005 | 0.004 | 0.004 | 0.003 | 0.006 | 0.007 | 0.007 | 0.007 | 0.007 | 0.008 | 0.008 | 0.007 | 0.007 | 0.007 | 0.006 | 0.006 | 0.006 | 0.006 | 0.006 | 0.006 | 0.006 | 0.006 | 0.006 | 0.006 | 0.005 | 0.005 | 0.005 | 0.005 | 0.005 | 0.004 | 0.004 | 0.004 | 0.004 | 0.004 | 0.004 |
| 7 | 0.000 | 0.018 | 0.018 | 0.013 | 0.013 | 0.017 | 0.014 | 0.014 | 0.013 | 0.012 | 0.011 | 0.010 | 0.012 | 0.012 | 0.012 | 0.012 | 0.011 | 0.012 | 0.012 | 0.009 | 0.008 | 0.008 | 0.008 | 0.009 | 0.008 | 0.008 | 0.009 | 0.009 | 0.008 | 0.009 | 0.009 | 0.008 | 0.008 | 0.008 | 0.008 | 0.008 | 0.008 | 0.008 | 0.008 | 0.007 | 0.007 | 0.007 | 0.007 |
| 8 | 0.000 | 0.000 | 0.009 | 0.009 | 0.011 | 0.016 | 0.014 | 0.013 | 0.013 | 0.011 | 0.010 | 0.010 | 0.009 | 0.008 | 0.008 | 0.007 | 0.007 | 0.007 | 0.007 | 0.007 | 0.006 | 0.006 | 0.007 | 0.007 | 0.007 | 0.007 | 0.007 | 0.007 | 0.007 | 0.006 | 0.006 | 0.005 | 0.006 | 0.005 | 0.005 | 0.005 | 0.005 | 0.005 | 0.005 | 0.005 | 0.004 | 0.004 | 0.004 |
| 9 | 0.000 | 0.000 | 0.018 | 0.018 | 0.016 | 0.018 | 0.015 | 0.014 | 0.014 | 0.012 | 0.011 | 0.011 | 0.014 | 0.014 | 0.013 | 0.013 | 0.012 | 0.011 | 0.010 | 0.011 | 0.011 | 0.011 | 0.011 | 0.011 | 0.011 | 0.011 | 0.011 | 0.011 | 0.011 | 0.010 | 0.009 | 0.008 | 0.008 | 0.008 | 0.008 | 0.008 | 0.008 | 0.007 | 0.007 | 0.007 | 0.006 | 0.006 | 0.006 |
| 10 | 0.000 | 0.018 | 0.009 | 0.009 | 0.009 | 0.006 | 0.004 | 0.004 | 0.004 | 0.004 | 0.003 | 0.003 | 0.003 | 0.003 | 0.003 | 0.003 | 0.003 | 0.003 | 0.003 | 0.003 | 0.003 | 0.003 | 0.003 | 0.003 | 0.003 | 0.003 | 0.003 | 0.003 | 0.003 | 0.003 | 0.003 | 0.003 | 0.003 | 0.003 | 0.003 | 0.003 | 0.003 | 0.002 | 0.002 | 0.002 | 0.002 | 0.002 | 0.002 |
| 11 | 0.000 | 0.000 | 0.000 | 0.004 | 0.007 | 0.008 | 0.006 | 0.006 | 0.005 | 0.005 | 0.004 | 0.004 | 0.003 | 0.003 | 0.003 | 0.003 | 0.003 | 0.004 | 0.004 | 0.003 | 0.003 | 0.003 | 0.003 | 0.003 | 0.003 | 0.003 | 0.003 | 0.003 | 0.002 | 0.003 | 0.002 | 0.002 | 0.002 | 0.002 | 0.002 | 0.002 | 0.002 | 0.002 | 0.002 | 0.002 | 0.002 | 0.002 | 0.002 |
| 12 | 0.000 | 0.000 | 0.000 | 0.004 | 0.009 | 0.013 | 0.010 | 0.010 | 0.009 | 0.008 | 0.007 | 0.006 | 0.008 | 0.007 | 0.007 | 0.007 | 0.006 | 0.007 | 0.007 | 0.007 | 0.007 | 0.007 | 0.007 | 0.007 | 0.007 | 0.006 | 0.007 | 0.006 | 0.006 | 0.006 | 0.006 | 0.005 | 0.005 | 0.005 | 0.005 | 0.005 | 0.005 | 0.005 | 0.004 | 0.004 | 0.004 | 0.004 | 0.004 |
| **Average** | **0.000** | **0.006** | **0.009** | **0.009** | **0.011** | **0.012** | **0.010** | **0.010** | **0.009** | **0.008** | **0.007** | **0.007** | **0.008** | **0.008** | **0.007** | **0.007** | **0.007** | **0.007** | **0.007** | **0.007** | **0.007** | **0.006** | **0.006** | **0.006** | **0.006** | **0.006** | **0.006** | **0.006** | **0.006** | **0.006** | **0.006** | **0.005** | **0.005** | **0.005** | **0.005** | **0.005** | **0.005** | **0.004** | **0.004** | **0.004** | **0.004** | **0.004** | **0.004** |
| Standard deviation | 0.000 | 0.009 | 0.007 | 0.004 | 0.003 | 0.004 | 0.004 | 0.003 | 0.003 | 0.003 | 0.003 | 0.003 | 0.004 | 0.004 | 0.004 | 0.003 | 0.003 | 0.003 | 0.003 | 0.003 | 0.003 | 0.002 | 0.003 | 0.003 | 0.003 | 0.002 | 0.003 | 0.003 | 0.003 | 0.002 | 0.002 | 0.002 | 0.002 | 0.002 | 0.002 | 0.002 | 0.002 | 0.002 | 0.002 | 0.002 | 0.002 | 0.002 | 0.002 |
| Standard error | 0.000 | 0.003 | 0.002 | 0.001 | 0.001 | 0.001 | 0.001 | 0.001 | 0.001 | 0.001 | 0.001 | 0.001 | 0.001 | 0.001 | 0.001 | 0.001 | 0.001 | 0.001 | 0.001 | 0.001 | 0.001 | 0.001 | 0.001 | 0.001 | 0.001 | 0.001 | 0.001 | 0.001 | 0.001 | 0.001 | 0.001 | 0.001 | 0.001 | 0.001 | 0.001 | 0.001 | 0.001 | 0.001 | 0.001 | 0.001 | 0.000 | 0.000 | 0.000 |

| **H2 gas generation rate(mL/㎠/hr)**  **PEO-Coated(N=12)** | | | | | | |  |  |  |  |  |  |  |  |  |  |  |  |  |  |  |  |  |  |  |  |  |  |  |  |  |  |  |  |  |  |  |  |  |  |  |  |  |
| --- | --- | --- | --- | --- | --- | --- | --- | --- | --- | --- | --- | --- | --- | --- | --- | --- | --- | --- | --- | --- | --- | --- | --- | --- | --- | --- | --- | --- | --- | --- | --- | --- | --- | --- | --- | --- | --- | --- | --- | --- | --- | --- | --- |
|  | 0.5 | 1 | 2 | 4 | 8 | 24 | 48 | 72 | 96 | 120 | 144 | **168** | 240 | 264 | 288 | 312 | 336 | 408 | 432 | 456 | 480 | **504** | 576 | 600 | 648 | 672 | 744 | 792 | **840** | 912 | 984 | **1104** | 1128 | 1152 | 1176 | 1296 | 1320 | 1440 | 1488 | 1632 | 1752 | 1776 | 1800 |
| 1 | 0.000 | 0.000 | 0.000 | 0.000 | 0.000 | 0.000 | 0.002 | 0.003 | 0.004 | 0.004 | 0.004 | 0.004 | 0.006 | 0.006 | 0.006 | 0.006 | 0.006 | 0.006 | 0.006 | 0.005 | 0.005 | 0.005 | 0.005 | 0.005 | 0.005 | 0.005 | 0.006 | 0.006 | 0.006 | 0.006 | 0.006 | 0.005 | 0.005 | 0.006 | 0.005 | 0.005 | 0.005 | 0.005 | 0.005 | 0.005 | 0.005 | 0.005 | 0.005 |
| 2 | 0.000 | 0.000 | 0.000 | 0.000 | 0.000 | 0.000 | 0.002 | 0.003 | 0.004 | 0.004 | 0.005 | 0.005 | 0.006 | 0.006 | 0.006 | 0.006 | 0.006 | 0.007 | 0.007 | 0.007 | 0.007 | 0.006 | 0.007 | 0.007 | 0.007 | 0.007 | 0.007 | 0.007 | 0.007 | 0.007 | 0.007 | 0.006 | 0.007 | 0.007 | 0.006 | 0.006 | 0.006 | 0.006 | 0.006 | 0.006 | 0.006 | 0.006 | 0.006 |
| 3 | 0.000 | 0.000 | 0.000 | 0.000 | 0.000 | 0.000 | 0.000 | 0.000 | 0.001 | 0.002 | 0.001 | 0.001 | 0.002 | 0.002 | 0.002 | 0.002 | 0.002 | 0.002 | 0.002 | 0.004 | 0.004 | 0.004 | 0.004 | 0.004 | 0.003 | 0.003 | 0.003 | 0.003 | 0.003 | 0.003 | 0.003 | 0.002 | 0.002 | 0.002 | 0.002 | 0.002 | 0.002 | 0.002 | 0.002 | 0.002 | 0.002 | 0.002 | 0.002 |
| 4 | 0.000 | 0.000 | 0.000 | 0.000 | 0.000 | 0.000 | 0.002 | 0.003 | 0.004 | 0.004 | 0.004 | 0.004 | 0.007 | 0.007 | 0.007 | 0.007 | 0.007 | 0.008 | 0.007 | 0.007 | 0.007 | 0.007 | 0.007 | 0.007 | 0.007 | 0.007 | 0.007 | 0.007 | 0.007 | 0.007 | 0.007 | 0.007 | 0.007 | 0.007 | 0.007 | 0.007 | 0.007 | 0.006 | 0.006 | 0.006 | 0.006 | 0.006 | 0.006 |
| 5 | 0.000 | 0.000 | 0.000 | 0.000 | 0.000 | 0.000 | 0.002 | 0.002 | 0.003 | 0.003 | 0.003 | 0.003 | 0.004 | 0.004 | 0.004 | 0.004 | 0.004 | 0.004 | 0.003 | 0.004 | 0.004 | 0.004 | 0.004 | 0.003 | 0.003 | 0.003 | 0.004 | 0.003 | 0.003 | 0.003 | 0.003 | 0.003 | 0.003 | 0.003 | 0.003 | 0.003 | 0.002 | 0.002 | 0.002 | 0.002 | 0.002 | 0.002 | 0.002 |
| 6 | 0.000 | 0.000 | 0.000 | 0.000 | 0.000 | 0.000 | 0.001 | 0.001 | 0.003 | 0.003 | 0.003 | 0.003 | 0.006 | 0.006 | 0.005 | 0.005 | 0.005 | 0.005 | 0.005 | 0.006 | 0.006 | 0.005 | 0.005 | 0.005 | 0.005 | 0.005 | 0.005 | 0.004 | 0.004 | 0.004 | 0.004 | 0.003 | 0.003 | 0.003 | 0.004 | 0.003 | 0.003 | 0.003 | 0.003 | 0.003 | 0.003 | 0.003 | 0.003 |
| 7 | 0.000 | 0.000 | 0.000 | 0.000 | 0.000 | 0.000 | 0.001 | 0.001 | 0.002 | 0.001 | 0.001 | 0.001 | 0.002 | 0.001 | 0.001 | 0.001 | 0.001 | 0.001 | 0.001 | 0.004 | 0.004 | 0.005 | 0.004 | 0.004 | 0.004 | 0.003 | 0.003 | 0.003 | 0.003 | 0.003 | 0.003 | 0.002 | 0.002 | 0.002 | 0.002 | 0.002 | 0.002 | 0.002 | 0.002 | 0.002 | 0.002 | 0.002 | 0.002 |
| 8 | 0.000 | 0.000 | 0.000 | 0.000 | 0.000 | 0.000 | 0.003 | 0.004 | 0.006 | 0.006 | 0.006 | 0.006 | 0.008 | 0.008 | 0.008 | 0.008 | 0.008 | 0.007 | 0.007 | 0.007 | 0.007 | 0.007 | 0.008 | 0.008 | 0.007 | 0.007 | 0.007 | 0.007 | 0.007 | 0.007 | 0.007 | 0.007 | 0.007 | 0.007 | 0.007 | 0.006 | 0.006 | 0.006 | 0.006 | 0.006 | 0.006 | 0.006 | 0.006 |
| 9 | 0.000 | 0.000 | 0.000 | 0.000 | 0.000 | 0.000 | 0.001 | 0.003 | 0.004 | 0.004 | 0.004 | 0.004 | 0.007 | 0.007 | 0.007 | 0.007 | 0.007 | 0.007 | 0.007 | 0.005 | 0.005 | 0.006 | 0.006 | 0.005 | 0.005 | 0.005 | 0.005 | 0.005 | 0.005 | 0.005 | 0.005 | 0.005 | 0.005 | 0.005 | 0.005 | 0.005 | 0.005 | 0.005 | 0.005 | 0.005 | 0.005 | 0.005 | 0.005 |
| 10 | 0.000 | 0.000 | 0.000 | 0.000 | 0.000 | 0.000 | 0.001 | 0.001 | 0.002 | 0.002 | 0.002 | 0.002 | 0.009 | 0.009 | 0.008 | 0.007 | 0.007 | 0.007 | 0.007 | 0.006 | 0.006 | 0.006 | 0.005 | 0.005 | 0.005 | 0.005 | 0.005 | 0.004 | 0.004 | 0.004 | 0.004 | 0.003 | 0.003 | 0.003 | 0.003 | 0.003 | 0.003 | 0.003 | 0.003 | 0.003 | 0.002 | 0.002 | 0.002 |
| 11 | 0.000 | 0.000 | 0.000 | 0.000 | 0.000 | 0.000 | 0.001 | 0.001 | 0.002 | 0.002 | 0.002 | 0.002 | 0.003 | 0.003 | 0.003 | 0.003 | 0.003 | 0.003 | 0.003 | 0.004 | 0.003 | 0.003 | 0.003 | 0.003 | 0.003 | 0.003 | 0.004 | 0.004 | 0.004 | 0.003 | 0.003 | 0.003 | 0.003 | 0.003 | 0.003 | 0.003 | 0.003 | 0.003 | 0.003 | 0.002 | 0.002 | 0.002 | 0.002 |
| 12 | 0.000 | 0.000 | 0.000 | 0.000 | 0.000 | 0.000 | 0.002 | 0.003 | 0.004 | 0.004 | 0.004 | 0.004 | 0.006 | 0.006 | 0.006 | 0.006 | 0.006 | 0.007 | 0.007 | 0.007 | 0.006 | 0.006 | 0.007 | 0.007 | 0.006 | 0.006 | 0.007 | 0.007 | 0.006 | 0.006 | 0.006 | 0.006 | 0.006 | 0.006 | 0.006 | 0.006 | 0.006 | 0.006 | 0.006 | 0.005 | 0.005 | 0.005 | 0.005 |
| **Average** | **0.000** | **0.000** | **0.000** | **0.000** | **0.000** | **0.000** | **0.002** | **0.002** | **0.003** | **0.003** | **0.003** | **0.003** | **0.005** | **0.005** | **0.005** | **0.005** | **0.005** | **0.005** | **0.005** | **0.006** | **0.005** | **0.005** | **0.005** | **0.005** | **0.005** | **0.005** | **0.005** | **0.005** | **0.005** | **0.005** | **0.005** | **0.004** | **0.005** | **0.005** | **0.005** | **0.004** | **0.004** | **0.004** | **0.004** | **0.004** | **0.004** | **0.004** | **0.004** |
| Standard deviation | 0.000 | 0.000 | 0.000 | 0.000 | 0.000 | 0.000 | 0.001 | 0.001 | 0.001 | 0.001 | 0.001 | 0.002 | 0.002 | 0.002 | 0.002 | 0.002 | 0.002 | 0.002 | 0.002 | 0.001 | 0.001 | 0.001 | 0.002 | 0.002 | 0.002 | 0.002 | 0.002 | 0.002 | 0.002 | 0.002 | 0.002 | 0.002 | 0.002 | 0.002 | 0.002 | 0.002 | 0.002 | 0.002 | 0.002 | 0.002 | 0.002 | 0.002 | 0.002 |
| Standard error | 0.000 | 0.000 | 0.000 | 0.000 | 0.000 | 0.000 | 0.000 | 0.000 | 0.000 | 0.000 | 0.000 | 0.000 | 0.001 | 0.001 | 0.001 | 0.001 | 0.001 | 0.001 | 0.001 | 0.000 | 0.000 | 0.000 | 0.000 | 0.000 | 0.000 | 0.000 | 0.000 | 0.000 | 0.000 | 0.001 | 0.001 | 0.001 | 0.001 | 0.001 | 0.001 | 0.001 | 0.001 | 0.001 | 0.001 | 0.001 | 0.000 | 0.000 | 0.000 |
